# Older adults’ experiences with using information and communication technology and tech support services in New York City: findings and recommendations for post-pandemic digital pedagogy for older adults

**DOI:** 10.3389/fpsyg.2023.1129512

**Published:** 2023-04-17

**Authors:** Ruth Finkelstein, Yiyi Wu, Mark Brennan-Ing

**Affiliations:** Brookdale Center for Healthy Aging, Hunter College, City University of New York, New York, NY, United States

**Keywords:** older adults, aging, information and communication technology, technology support, technology training for older adults, COVID-19, survey research

## Abstract

**Introduction:**

Although Information and Communication Technology (ICT) has great potential to help older adults cope with challenges associated with aging, the intended benefits of ICT are not always realized in this population due to access barriers and low digital literacy. During the COVID-19 pandemic, numerous tech support initiatives for older adults got underway. However, evaluation of the effectiveness of these initiatives is less common. This research partnered with a large, multi-service organization in New York City that gave some groups of their clients ICT devices, unlimited broadband, and access to technology training in response to COVID-19 lockdowns. This study investigates older adults’ experiences with ICT and ICT support services to better inform the existing and emerging tech support for older adults during and beyond the pandemic.

**Methods:**

Data were obtained from interviewer-administered surveys of 35 older adult recipients of ICT devices, connectivity, and training in New York City. The average age was 74 years (range = 55–90 years). The group was diverse regarding race/ethnicity (Black 29%, Latino 19%, White 43%). All had low incomes. Surveys consisted of multiple-choice items and open-ended responses.

**Results:**

The study found that one size does not fit all when it comes to ICT training and support for older adults. While connection to devices and services and tech support led to a degree of ICT adoption, the newly learned skills did not always lead to expanded device usage. The readily available tech support training and support do not guarantee service utilization, as success with tech services is related to one’s pre-existing ICT competence.

**Discussion:**

The study concludes that customized training based on individuals’ skills rather than age is needed. Tech support training should start by understanding an individual’s interests and incorporate tech education to help users identify a wide range of existing and emerging online services that can meet their needs. Service organizations should consider including an assessment of ICT access, use, and skills into their standard intake protocols to ensure effective service delivery.

## Introduction

Information and communication technology (ICT) use was necessary to stay socially connected and capable of receiving many health and social services during the COVID-19 pandemic shutdowns. ICT access was particularly important for older adults who were under the most stringent isolation protocols (Llorente-Barroso et al., 2021). While early reports had optimistic projections of older adults’ adoption and ownership of ICT over the years, especially during the course of the pandemic (Kakulla, 2021; Faverio, 2022), many studies warned that such growth often only represented a shift from “have nots” to “haves” (Freeman et al., 2022). In other words, the mere possession of ICT devices is not enough to guarantee meaningful digital access and engagement. Compared to younger adults, older adults tend to have overall lower digital literacy and less success in efficiently achieving their goals and accurately addressing their needs as a result of internet usage (Van Deursen, 2020).

It is crucial to treat older adults not as a monolithic group of technologically-incompetent users. Older adults possess a wide range of digital skills and engage with ICT in varying ways (Van Deursen and Helsper, 2015a; Hänninen et al., 2020). However, the digital divide between older and younger adults in infrastructural access and digital literacy still remains (Smith, 2014; Hecker et al., 2021; Perrin and Atske, 2021). The 2022 Pew Research Center survey found that 86% of those ages 30–64 owned broadband access at home compared to 61% of those ages 65 and older (Faverio, 2022). Among the older adults who are connected, despite the subset of older adults who are proficient in technology use, compared to the younger population, the overall older population is at lower odds of integrating internet use into everyday activities and uses the internet in more limited ways owing to lower literacy, inability to see the benefits of online engagement, and, sometimes, genuine disinterests in using the internet (Quan-Haase et al., 2018). Some older adults reported being overwhelmed by the variety of ICT functions and struggling with a lack of clear instructions and adequate support, which resulted in an inability to expand usage and increasing frustration with learning new things (Vaportzis et al., 2017; Harris et al., 2022). As a result of rapid digitalization and persistent issues related to the digital divide, some older adults had to give up on certain activity engagements as these activities became no longer available offline (Reneland-Forsman, 2018).

The digital divide became even more visible as the US service organizations transitioned overnight to remote service delivery in March 2020. Many older adults faced obstacles and steep learning curves. Although the pandemic has pushed many older adults to use ICT to some extent to communicate with social networks and keep up with information, many functions and applications of ICT remain poorly utilized and understood. Reports on older adults’ digital engagement during the pandemic have found significantly less ICT usage among older adults to manage daily activities and access health services (Lam et al., 2020; Kakulla, 2021; Perrin and Atske, 2021). In the same 2021 AARP Tech Trends report that highlighted older adults’ new tech purchases during the pandemic, more than half of the surveyed older adults (*n* = 2,271) reported needing additional support with their purchased devices and almost 40 percent of them lacked digital confidence (Kakulla, 2021).

Meanwhile, socio-demographic factors also impact older adults’ internet usage. Declining internet usage has been associated with growing age and those above age 75 are less likely to access the internet than those from the younger group of older adults (65–74) (Smith, 2014; Crouch and Gordon, 2019; Sixsmith et al., 2022). Studies have also found that adults with higher educational status in early life were more likely to engage in internet use in later life (Leukel et al., 2021). In addition, ICT adoption patterns are also influenced by an individuals’ physical and cognitive capability. Visual and hearing impairment and memory loss in older adults are associated with decreased usage of the internet (Gell et al., 2015; Choi et al., 2020; Lam et al., 2020). All the existing studies and reports have underlined the need to implement appropriate and adequate tech support and training for older adults.

Previous researchers have explored older adults’ ICT learning technology support needs and learning preferences. Recognizing a wide range of skill levels and needs across individuals, studies identified a demand for personalized support that fulfills a broad range of interests (Barnard et al., 2013; Hunsaker et al., 2019; Schlomann et al., 2022). Older adults with sufficient internet skills may not need tech support or emphasize getting the support that expands their ICT abilities to engage in a broader range of digital activities (Quan-Haase et al., 2018; Hunsaker et al., 2019). To older users who are more dependent on others’ support, the availability and immediacy of help is a critical matter (Hunsaker et al., 2019). While formal technology support and training is also identified as one of the technical support sources among older adults, due to concerns related to cost and accessibility, many reported getting help from informal sources such as their families and friends who are known as “warm experts” (Lafontaine and Sawchuk, 2015; Hunsaker et al., 2019; Hänninen et al., 2020). Introduced by sociologist Bakardjieva (2005), the term warm expert refers to more technologically experienced individuals who are in a tech novice’s close social network, who help inexperienced tech users to solve their technical problems and contribute to their learning process. To older adults, ‘warm experts’ typically are their close personal networks, such as families, friends, peers, and social service providers, who are able to offer personalized support in a timely manner (Olsson and Viscovi, 2018; Hänninen et al., 2020; Hunsaker et al., 2020; Nordin et al., 2021). Moreover, as indicated by Quan-Haase et al.’s (2018) study, tech support might not be desired by all older adults with limited skills and experiences, as some place more value on offline engagements and perceive the tech learning process as a waste of time and effort.

Regarding device preferences, some older adults find it easier to use tablets than mobile phones or laptops, as tablets provide larger screens than regular phones but allow more flexibility than computers (Chan et al., 2016). Likewise, smart (i.e., internet capable) TVs have been identified as appropriate and effective learning tools for older adults because TVs provide a familiar and easy-to-use interface for the older population, helping to minimize technology resistance and anxiety (Santana-Mancilla and Anido-Rifón, 2017; Andreadis et al., 2021; Wang and Wu, 2021).

As shown, past research has well demonstrated older adults’ heterogenous digital engagement patterns, infrastructural access and narratives on preferences in adoption. However, there is insufficient research that extensively and empirically interrogates older adults’ experiences with an existing tech support service. While many studies interrogating older adults’ support needs took place before the COVID-19 crisis, the arrival of the pandemic has changed the techno-social environments for many as the integration of ICT use into everyday life has become more necessary than ever. Meanwhile, throughout the pandemic, the importance of technology support services for older adults has been highlighted as health and community-based organizations pivoted to virtual service delivery and programming. Numerous efforts have been made by both the public and private sectors to support older ICT learners. Nevertheless, the effectiveness of these initiatives has not been examined for the most part. In this study, we have an opportunity to examine older adults’ real-life experiences and behaviors with regard to tech support usage during the pandemic in a small sample. This study takes advantage of a setting where material access problems were solved (as people were provided free devices and broadband connection) and available training and tech support services were provided, to ask what are the further barriers and facilitators to older adults’ effective usage of ICT during the pandemic and beyond?

In this study we evaluate the effectiveness of ICT support through the meaningful access framework. In the past decade, the digital inclusion/exclusion framework had gradually moved from a broad discussion of the differences in physical access to a nuanced recognition of differences in attitudes, types of engagement, skill levels, and the tangible outcomes as result of ICT usage (Witte and Mannon, 2010; Van Deursen and Helsper, 2015b). This framework conceptualizes three levels of digital divide. The first level concerns disparities in infrastructural access; the second level focuses on gaps in skill levels, digital literacy and uses; and the third level assesses the differences in the impact generated by internet use (Van Deursen and Dijk, 2014; Van Deursen and Helsper, 2015b). Translating such theoretical framework to the policy work focusing on ICT and older adults, the Brookdale Center for Healthy Aging identified four essential components that constitute meaningful digital access for older adults: a usable device, adequate broadband internet service, the education to foster skills, and ongoing technical support to ensure one’s capability to navigate the internet independently to meet one’s needs (González-Rivera and Finkelstein, 2021). Consistent with the framework for digital inclusion, Brookdale’s meaningful access concerns not only the basic infrastructural capabilities or possession of basic skills to go online but also one’s capacity to make use of the internet independently and benefit from everyday digital engagement. Therefore, successful education and tech support should result in knowledge about the usefulness of ICT including its specific functions and platforms that fuel motivation, and effective performance of online activities that ensure meaningful access and realize the intended benefits of ICT for older adults (González-Rivera and Finkelstein, 2021).

We partnered with a large organization serving older adults in New York City which had launched an ICT enhancement initiative for its clients in response to the COVID-19 pandemic. The specific group receiving these devices included all the residents of one independent living apartment building for older adults; participants from a chronic disease self-management program; clients of mental health clinic; participants from a palliative care program; and participants from several senior centers (voluntary recreation and meal sites for older adults living in the community). All of them were connected to a variety of resources and stably housed. Following the COVID-19 lockdown, this organization provided groups of their clients ICT devices, including laptops, tablets, and TV set top boxes that connected to the internet (i.e., smart TVs). Clients did not have their own choice of device; different devices were available in different settings. However, in all instances, this initiative included installation assistance, user manuals, free unlimited broadband, and ongoing technology training and support services provided by two tech support organizations who were experienced in working with older adults. Additionally, clients were able to receive support from the service organization’s staff members whom they knew well, which facilitated self-paced learning. We evaluated the impact of this effort and explored older adults’ experiences with ICT support services. Given the combination of removal of infrastructural barriers (devices, set up, connection all provided) and provision of training and ongoing support, this initiative offered a useful opportunity to investigate older adults’ engagement with ICT devices and the efficacy of ICT technology support. This study aimed to: (1) better understand the technology competence, use, and barriers of older tech service recipients who participated in this program; (2) provide guidance about the impact of various components including the use of specific devices, connectivity, training, and support services; (3) evaluate the strengths and weaknesses of the organization’s tech access programs; (4) ultimately, present evidence useful for the future creation and expansion of technology services for older adults. We attempt to address gaps in the current literature by exploring not only older adults’ interactions with internet-connected devices but also, specifically, their experiences with ICT support services.

## Materials and methods

### Service program specifics

The organization distributed internet-capable devices and broadband access to five groups of program participants. The specific details of the programs are presented below:

• Wired a senior residence for wifi and provided residents with a laptop and tech support services. Tech support services included initial device installation, lessons, and ongoing remote services provided by the company’s staff specializing in services for older adults to support participants’ tech use whenever problems arise. Services were provided in English, Russian, and Hebrew.
• Provided tablets and tech support services to participants in three of the organization’s service programs: mental health, chronic disease self-management, and palliative care.Tech support services were the same as for people in the residence.
• Distributed TV set top adapters for internet connection to senior center participants to enable access to various interactive virtual programming, including fitness classes, peer networking, and professional development sessions. Tech support was offered by the provider of the adapters, and included installation and ongoing tech support through a helpline.

### Data collection procedures

This evaluation utilized a survey research design. Because of the patient confidentiality regulations at the studied research site, our researchers were not allowed to recruit participants directly but through the support of the service organization’s staff members. While the number of participants reached during the recruitment process was unknown, a total of 35 older adults reached out to our researchers via a given contact number and were interviewed. Additionally, we communicated with the two tech services providers, program leads and the sponsoring organization’s site staff directly, which supplemented the data collection effort. The program evaluation protocol was approved by the City University of New York Institutional Review Board.

To maintain client confidentiality, we used a passive recruitment strategy. Data collection took place primarily between January 2022 and March 2022. Based on clients’ demographic information provided by the older adult’s organization and within the linguistic capacity of our research team, we sent flyers in English, Russian, and Spanish to the five respective program leads, who then distributed the recruitment flyer to all program participants who had received devices. Interested clients called a number provided on the flyer to schedule 30-minute interviews by phone. Prior to the interview, respondents provided verbal informed consent and were guaranteed anonymity and confidentiality of their information. Interview data were recorded in Qualtrics survey software. Non-English speakers had the option to be interviewed in Spanish or Russian, but all interviews were conducted in English. Respondents were compensated for their time with $20 gift cards.

### Data collection instrument

The questionnaire was organized into six domains: demographics, self-assessed wellness, social connection, technology use, technology support, and technology attitudes. In addition to providing personal baseline information, including health status and familiarity with computers, respondents were asked to:

• Assess change in contact and feelings of closeness with their social networks compared to before COVID-19, change in using technology to connect with social networks since COVID-19.
• Rate the frequency and ease of use of internet-connected devices (tablets, TV boxes).
• Describe the assistance and support provided by the tech providers, when relevant.
• Describe their experiences with the devices, as well as the impact those devices have had on their daily lives.
• Assess their level of skill and confidence in conducting various online activities.
• Assess their satisfaction with participating in the program and importance of an internet connection during the pandemic.
• Assess loneliness using the 3-item version of the UCLA Loneliness Scale (Russell, 1996), a commonly used measure for older adults. The UCLA Scale demonstrated high internal consistency in this sample (Cronbach’s alpha = 0.88).

The majority of questionnaire items used categorical or Likert-type scale responses. However, each section also included open-ended questions, including items specific to experiences with the laptop, tablet, and TV set-top devices, as well as the initial installation, training, ongoing support, and barriers to/facilitators of device utilization to allow for a greater depth of information.

## Findings

### Description of the sample

We recruited a diverse sample of participants with regard to age, race/ethnicity, and physical and mental health conditions. Almost half of the participants (16) were between 70 and 79 years old, and 8 participants were 80 years of age or older [see Table 1]. More than half of the participants were Black/African Americans and Latinx. Since the sponsoring organization serves low-income older adults, participants were low-income. However, no one was precariously or unstably housed as the organization provides housing and/or housing support.

**TABLE 1:** Program details and demographic characteristics tech recipient user type.

			Total					
			***N***			**%**		
Senior residence–chromebook			7			20.6		
Mental health program–tablet			9			26.5		
Chronic disease management–tablet			6			17.6		
Senior center–TV box			12			35.3		
	**Total**		**Experienced**		**New adopter**		**No mastery**	
	***N***	**%**	***N***	**%**	***N***	**%**	***N***	**%**
**Age group**								
55–69	9	27.3	4	40.0	4	26.7	1	12.5
70–79	16	48.5	2	20.0	8	53.3	6	75.0
80–90	8	24.2	4	40.0	3	20.0	1	12.5
**Gender**								
Female	28	82.4	8	80.0	13	81.3	7	87.5
Male	6	17.6	2	20.0	3	18.8	1	12.5
**Race/ethnicity**								
Black	12	37.5	4	40.0	6	37.5	2	33.3
White	11	34.4	6	60.0	4	25.0	1	16.7
Hispanic	6	18.8	0	0.0	4	25.0	2	33.3
Other	3	9.4	0	0.0	2	12.5	1	16.7
**Marital status**								
Single	6	18.8	2	22.2	1	6.3	3	42.9
Married/partner	5	15.6	2	22.2	3	18.8	0	0.0
Divorced/separated	5	15.6	2	22.2	3	18.8	0	0.0
Widowed	16	50.0	3	33.3	9	56.3	4	57.1

Lives alone	25	75.8	8	80.0	12	75.0	5	71.4
Retired	32	94.1	9	90.0	16	100.0	7	87.5
**Last job**								
Unskilled	1	3.0	1	10.0	0	0.0	0	0.0
Skilled	12	36.4	3	30.0	7	46.7	2	25.0
Professional/managerial	10	30.3	2	20.0	5	33.3	3	37.5
Proprietor	8	24.2	3	30.0	3	20.0	2	25.0
Volunteer	1	3.0	1	10.0	0	0.0	0	0.0
Never worked	1	3.0	0	0.0	0	0.0	1	12.5
Used tech last job	15	44.1	4	40.0	7	43.8	4	50.0

Experienced *N* = 10. New adopter *N* = 16. No mastery *N* = 8. CDSM, chronic disease self-management. Only valid responses shown.

A majority of participants (*n* = 22) reported trouble using their hands due to arthritis. Nearly half (*n* = 14) reported trouble seeing even with glasses or contact lenses, and 7 had difficulty hearing even if using a hearing aid. No one reported severe cognitive declines while a few (*n* = 8) reported having somewhat poor memory. A majority of participants reported that their daily activities had either been severely restricted (*n* = 2) or somewhat restricted (*n* = 19) by their physical health. In terms of mental health, while most participants reported overall good mental wellbeing, two reported that their mental health had severely affected their ability to perform daily activities. Despite repeated attempts and substantial effort by the organization’s program staff, no clients from the palliative care program answered the survey. Program staff provided their perspectives on the reasons for this lack of response, which are incorporated into findings and practical implications.

### Resolved infrastructural access barrier leads to a degree of ICT adoption

Overall, most participants already had or attained some proficiency through the technology support programs for at least some ICT functions. Those individuals shared rather positive attitudes toward ICT use and reported that using the internet was very important during the pandemic (*n* = 30) and that their experiences with ICT devices and support services were good or very good (*n* = 31). One person shared that, “It’s unbelievable what that monster can do. I can’t live without it. It’s the center of my life.”

Free devices and the internet are shown to be one of the facilitators for participants to adopt ICT. One person stated: “I take full advantage of it. It was given to me as a gift and it would be selfish not to use it.” Another participant who previously did not have a device expressed her joy upon receiving her tablet: “I always wanted one. And [the organization’s name] gave me one!” Although this participant still struggled to use most of the device functions, the participant learned how to send emails and use zoom to take guitar classes. As the first-level of the digital divide was solved through free devices and broadband, some individuals who were initially hesitant about using devices became more open to the idea of ICT use. One person shared: “I stopped using it at the beginning. But then I thought, they took all this energy to give me this thing, I’ll give it another try…I’ve been very happy with the online offerings. It has enhanced my life a lot.” To some, it was the combination of the free devices and the impact of COVID-19 that made them appreciate the ICT utilization: “Before covid, it was better. We would go to the theater, museums, and concerts. Now, the world is more narrow. But the computer helps expand it.”

These findings are consistent with several studies, suggesting strongly that the belief that most older adults lack the ability and desire to learn ICTs is simply not accurate (Vaportzis et al., 2017; Sixsmith et al., 2022). Older adults are capable of improving their life through ICT usage with appropriate support (Francis et al., 2019). The design of this tech-enhancement program—eliminating basic infrastructural barriers– supports the explanation that much non-use of ICT by older adults stems from a lack of infrastructural access rather than a lack of interest. At the same time, material access issues, such as concerns with the cost of devices and the internet, affect many digitally marginalized communities regardless of age.

### Health characteristics in dialog with tech usage

While past research revealed a significant association between physical and mental health challenges and reduced technology use among the older population (Smith, 2014), we found a more complicated relationship between health and tech use after the access barriers were removed [see Table 2]. There were a total of 8 infrequent users/non-users among the 35 people surveyed who reported no or very infrequent use of their devices. Interestingly, while the prevalence of respondents reporting either difficulty seeing, hearing, or using their hands was high among participants (41, 21, and 65%, respectively), they were NOT overrepresented in the group who did not adopt devices. Such a non-associative relationship between tech use and health conditions further shows that once the access barriers are solved, the impact of health-related barriers is mitigated.

**TABLE 2:** Health characteristics by tech recipient user type.

	Total		Experienced		New adopter		No mastery	
	***N***	**%**	***N***	**%**	***N***	**%**	***N***	**%**
Trouble seeing	14	41.2	4	40.0	7	43.8	3	37.5
Trouble hearing	7	20.6	4	40.0	2	12.5	1	12.5
Trouble using hands	22	64.7	6	60.0	11	68.8	5	62.5
**Self-rated memory**								
Very bad	0	0.0	0	0.0	0	0.0	0	0.0
Somewhat bad	3	8.8	0	0.0	2	12.5	1	12.5
Neither good nor bad	10	29.4	3	30.0	4	25.0	3	37.5
Somewhat good	15	44.1	5	50.0	8	50.0	2	25.0
Very good	6	17.6	2	20.0	2	12.5	2	25.0
**Self-rated health**								
Very bad	1	2.9	0	0.0	0	0.0	1	12.5
Somewhat bad	2	5.9	0	0.0	1	6.3	1	12.5
Neither good nor bad	11	32.4	4	40.0	5	31.3	2	25.0
Somewhat good	15	44.1	5	50.0	8	50.0	2	25.0
Very good	5	14.7	1	10.0	2	12.5	2	25.0
**Self-rated quality-of-life**								
Very bad	1	3.0	0	0.0	0	0.0	1	12.5
Somewhat bad	2	6.1	1	10.0	1	6.7	0	0.0
Neither good nor bad	4	12.1	1	10.0	1	6.7	2	25.0
Somewhat good	15	45.5	3	30.0	9	60.0	3	37.5
Very good	11	33.3	5	50.0	4	26.7	2	25.0

Experienced *N* = 10. New adopter *N* = 16. No mastery *N* = 8. Only valid responses shown.

Nevertheless, although self-reported difficulty seeing, hearing, and using one’s hands did not hinder adoption, it does appear that there are states of disability, physical or mental, beyond which interest in or capacity for ICT is not present. Among the 8 infrequent users/non-users, there were 3 respondents who reported bad/very bad physical and/or emotional health. Half of them were very challenged during the tech enhancement program period, including one reporting having had suicidal thoughts, another who “lost” the use of device immediately and could find no help, and another who reported being unable to read and, therefore, unable to use the device^1^. The finding that some people find ICT adoption out of reach due to their health is further supported by our unsuccessful attempt to reach clients from the organization’s palliative care program. While a 2016 systematic review of ICT usage in palliative care pointed to the potential of ICT in aiding decision-making, the particular research field is small (Ostherr et al., 2016), with a sharp focus on ICT adoption from care providers’ perspectives than patients’ (Portz et al., 2020; Mills et al., 2021). Echoing many others, this research calls for more future research to continue exploring questions pertinent to adoption patterns among older palliative care patients.

### Utilization of tech support services is related to degree of ICT competency

The 35 people we surveyed were categorized into three groups based on their responses to questions about their technology use before the new devices were distributed and their responses to questions about functions, frequency, and ease of use of the new devices. People (*n* = 10) who already had ICT competence (most also already owned at least one device before the program) and then added use of the new device were categorized as “experienced.” Those (*n* = 17) who had little/no previous experience with ICT and gained the competence and confidence to do several functions on their new device and did so at least weekly were categorized as “new users.” Those who reported less than weekly use and no specific functions used (*n* = 8) were categorized as “infrequent users/non-users.”

About one-third of the respondents (*n* = 10) already had experience using an internet-connected device (desktop computer, tablet, laptop) before they received their new devices as part of the program. Not surprisingly, this group uniformly found the new devices easy/very easy to use. They were also the group that was the most likely to request and successfully receive training and technical assistance from the tech support companies. These respondents were typically happy to receive a new device and often reported using it differently than their existing one. They often described the tech support service as “available” and “helpful.” Many called tech support for troubleshooting whenever they encountered problems. One participant took advantage of the training class to expand their tech skills: “I already know how to use a computer. Also, [the organization’s name] gave me very good computer classes.” Another participant successfully acquired skills to set up and use their new tablet by contacting technology support services and even offered assistance to their peers: “[The name of the technology service] girls were very helpful. I taught myself based on what they said. Then I helped others, about 30 to 40 people in the building! I’d go house to house.”

Almost half the participants were the agency’s intended target for the ICT distribution and training program: older adults who did not have access to devices and the internet at the start of the pandemic and gained it through this initiative. Sixteen of the people interviewed who reported no previous experience with ICT were pleased with their tech-learning experiences. However, in contrast to the “experienced” group who benefited most from the tech support programs, most “new users” did not seek further support from the formal tech support program following the initial start up session. Of those who did, several reported that the tech support program was not helpful. As one participant summarized succinctly, “It’s all very simple to understand. We needed private tutors. How do you give computers to people who never used them without any basic education? Those who already owned computers benefited. Others, of course not.”

Most of the time, among the new users, consistent with previous research (Lafontaine and Sawchuk, 2015; Hunsaker et al., 2019; Hänninen et al., 2020), warm experts, including families, peers, and the organization’s social service staff, were the preferred sources of technical support. Although everyone we surveyed was aware of the existence of the professional tech support services, most reported not requesting any assistance from tech support. One participant shared the story of having their daughter contact the tech support for help.

### Inability to apply learned skills to expanded usage

While most respondents learned to use their devices, for many, the functions they used regularly were somewhat limited, and centered on the core functions the organization needed them to master in order to maintain the continuity of services during the COVID-19 lockdowns.

As shown in Table 3, the majority of the laptop and tablet recipients reported that they could do a suite of functions well or very well. These tasks included sending/receiving email, online shopping (though a significant minority —43% report not doing this at all), reading online publications, accessing educational and recreational sites (like senior centers), playing games, watching TV online, receiving video calls, communicating with a case manager or service provider, and taking classes online. The online functions that were less frequently endorsed included: online banking, accessing benefits information, accessing social media sites, participating in chats or blogs, and using telehealth services. There was a sizable minority who reported only using their device to interact with the service provider, like answering Zoom calls from the organization’s workers and participating in online classes offered by the organization. A few could do some online tasks, such as watching TV online and accessing zoom, but were unable to do other things like reading newspapers online and playing games.

**TABLE 3:** Self-rating of ability to do computer functions by tech recipient user type.

	Total		Experienced		New adopter		No mastery	
	***N***	**%**	***N***	**%**	***N***	**%**	***N***	**%**
**Send/receive email**								
Very well	12	36.4	8	80.0	3	18.8	1	14.3
Well	7	21.2	2	20.0	4	25.0	1	14.3
Fairly well	3	9.1	0	0.0	3	18.8	0	0.0
Poorly	2	6.1	0	0.0	2	12.5	0	0.0
Not at all	9	27.3	0	0.0	4	25.0	5	71.4
**Online shopping**								
Very well	12	36.4	6	60.0	5	31.3	1	14.3
Well	2	6.1	1	10.0	1	6.3	0	0.0
Fairly well	0	0.0	0	0.0	0	0.0	0	0.0
Poorly	1	3.0	0	0.0	0	0.0	1	14.3
Not at all	18	54.5	3	30.0	10	62.5	5	71.4
**Reading online pubs**								
Very well	12	36.4	4	40.0	7	43.8	1	14.3
Well	5	15.2	2	20.0	2	12.5	1	14.3
Fairly well	2	6.1	0	0.0	2	12.5	0	0.0
Poorly	1	3.0	0	0.0	0	0.0	1	14.3
Not at all	13	39.4	4	40.0	5	31.3	4	57.1
**Online banking**								
Very well	3	9.1	1	10.0	2	12.5	0	0.0
Well	5	15.2	3	30.0	2	12.5	0	0.0
Fairly well	3	9.1	2	20.0	1	6.3	0	0.0
Poorly	0	0.0	0	0.0	0	0.0	0	0.0
Not at all	22	66.7	4	40.0	11	68.8	7	100.0
**Accessing benefit information or benefit application**								
Very well	3	9.1	1	10.0	2	12.5	0	0.0
Well	3	9.1	2	20.0	1	6.3	0	0.0
Fairly well	6	18.2	4	40.0	2	12.5	0	0.0
Poorly	1	3.0	0	0.0	0	0.0	1	14.3
Not at all	20	60.6	3	30.0	11	68.8	6	85.7
**Accessing educational or recreational site**								
Very well	14	42.4	6	60.0	7	43.8	1	14.3
Well	3	9.1	1	10.0	2	12.5	0	0.0
Fairly well	2	6.1	0	0.0	0	0.0	2	28.6
Poorly	1	3.0	0	0.0	1	6.3	0	0.0
Not at all	13	39.4	3	30.0	6	37.5	4	57.1
**Playing games or downloading media**								
Very well	17	51.5	7	70.0	9	56.3	1	14.3
Well	2	6.1	1	10.0	1	6.3	0	0.0
Fairly well	3	9.1	1	10.0	1	6.3	1	14.3
Poorly	0	0.0	0	0.0	0	0.0	0	0.0
Not at all	11	33.3	1	10.0	5	31.3	5	71.4
	***N***	**%**	***N***	**%**	***N***	**%**	***N***	**%**
**Online phone/video calls**								
Very well	13	39.4	6	60.0	6	37.5	1	14.3
Well	9	27.3	2	20.0	6	37.5	1	14.3
Fairly well	3	9.1	1	10.0	0	0.0	2	28.6
Poorly	1	3.0	0	0.0	1	6.3	0	0.0
Not at all	7	21.2	1	10.0	3	18.8	3	42.9
**Social media sites**								
Very well	11	33.3	6	60.0	5	31.3	0	0.0
Well	2	6.1	2	20.0	0	0.0	0	0.0
Fairly well	3	9.1	1	10.0	1	6.3	1	14.3
Poorly	0	0.0	0	0.0	0	0.0	0	0.0
Not at all	17	51.5	1	10.0	10	62.5	6	85.7
**Chats/blogs/forums**								
Very well	1	3.0	0	0.0	1	6.3	0	0.0
Well	1	3.0	1	10.0	0	0.0	0	0.0
Fairly well	1	3.0	1	10.0	0	0.0	0	0.0
Poorly	0	0.0	0	0.0	0	0.0	0	0.0
Not at all	30	90.9	8	80.0	15	93.8	7	100.0
**Telehealth**								
Very well	5	15.2	1	10.0	4	25.0	0	0.0
Well	7	21.2	5	50.0	2	12.5	0	0.0
Fairly well	4	12.1	1	10.0	2	12.5	1	14.3
Poorly	0	0.0	0	0.0	0	0.0	0	0.0
Not at all	17	51.5	3	30.0	8	50.0	6	85.7
**Communicating with case manager/other helper**								
Very well	8	25.0	3	33.3	5	31.3	0	0.0
Well	8	25.0	3	33.3	4	25.0	1	14.3
Fairly well	0	0.0	0	0.0	0	0.0	0	0.0
Poorly	0	0.0	0	0.0	0	0.0	0	0.0
Not at all	16	50.0	3	33.3	7	43.8	6	85.7
**Participate in group activity or class**								
Very well	18	54.5	7	70.0	10	62.5	1	14.3
Well	3	9.1	0	0.0	2	12.5	1	14.3
Fairly well	2	6.1	1	10.0	1	6.3	0	0.0
Poorly	0	0.0	0	0.0	0	0.0	0	0.0
Not at all	10	30.3	2	20.0	3	18.8	5	71.4

Experienced *N* = 10. New adopter *N* = 16. No mastery *N* = 8. Only valid responses shown.

This suggests that many people mastered what they were explicitly taught, but are less inclined to venture further into the options/opportunities their devices provide or transfer the learned skills to perform other activities. This is consistent with our previous research that for people for whom ICT is new, it is not always evident what the device is useful for, even when some skills are gained (González-Rivera and Finkelstein, 2021).

## Discussion: moving forward

In examining older adults’ experiences with an existing tech support service during the pandemic, the majority of the findings of this study are consistent with past findings on older adults’ heterogenous internet use and engagement in the pre-pandemic era. The results of our study once again show that older adults possess a range of tech skills and needs that require different levels of training and support based on an individual’s skills and needs (Hunsaker et al., 2019; Schlomann et al., 2022).

Importantly, this study found that those with previous tech experiences and a degree of digital literacy benefited most from the tech support service. New users with limited tech experiences continue to prefer “warm experts” as their instructors. Some past studies ascribed the inability to participate in formal tech service support and training to a lack of availability, immediacy, affordability, and accessibility (Hunsaker et al., 2019; Hänninen et al., 2020). However, in this research, although all the participants were aware of the readily available professional tech support staff who offered free support, the new ICT users still more frequently sought support from families, friends, and their regular support providers who may not be the tech-teaching experts. A possible explanation could be that those with more tech knowledge are better at recognizing and articulating the specific problem they have encountered and the particular support they need (Hunsaker et al., 2019). Meanwhile, new learners may prefer informal learning environments and obtaining help from those they have close relationships with, which helps relieve tech novices’ stress and fear while learning new things (Lafontaine and Sawchuk, 2015).

The uneven utilization of ICT functions and applications may partly be the result of being taught “how to do this” without understanding “what this is good for.” In this specific program, the organization offered the devices and training in an emergency situation with the goal of continuing to keep older adults connected to their services during the pandemic. The people in this program learned the computer functions they were explicitly taught. Many of our informants did not recognize the implications for other uses from what they were taught. While such forms of tech support could be effective and appropriate in addressing older service recipients’ immediate needs during a time of crisis, it remains crucial for future tech support programs to create learning tools that allow older adults to translate learned skills to a broader range of online activities based on their needs and goals (Quan-Haase et al., 2018).

Our previous analysis of the New York City census re access to technology found predictors of internet access among older adults included younger ages, higher levels of formal education, higher income, and living with others (González-Rivera and Finkelstein, 2021). Other studies report that people in poorer physical, emotional, and cognitive health are less likely to adopt ICT (Crouch and Gordon, 2019; Leukel et al., 2021; Sixsmith et al., 2022). Among our informants, ICT adoption was not associated with younger age, higher education, and health conditions. While many of these participants were low-income and racial and ethnic minorities, all were clients of a large aging services organization and, therefore, were connected to a variety of resources and stably housed. Therefore, we understand this finding as supporting the idea that material access barriers and socioeconomic disadvantages may drive much of the lower uptake of ICT among older adults in the overall population in New York City.

Moreover, despite the infrastructure and technical support provided, some participants did not use any ICT and others used it in limited ways. Those who reported very limited usage and knowledge of ICT devices were in overall good health and were not miserable. To quote one participant: “I meditate and pray every day. I read spiritual material, do puzzles, keep myself busy and I don’t feel lonely. I draw and do math. I got the tablet in the mail, but don’t know how to use it.” Even during a time of crisis when technological competency was regarded as a determinant of quality of life, consistent with the previous findings before the pandemic, older participants did not let their tech skill levels impact their social involvement and exhibited a high level of autonomy in deciding the ways to live their lives (Quan-Haase et al., 2016, 2018).

### Tech support design and policy recommendations

Our findings have implications for program and policy. First, “purpose built” training may not optimize all the possible ways to access the many functions and range of online experiences enabled by these devices. It is important to incorporate tech education into the tech support services that can keep older people updated on the wide range of existing and emerging online services they could benefit from. As many existing studies have pointed out, the perceived usefulness of the internet is one of the motivators for internet use among older adults (Yap et al., 2022). Therefore, tech support training should start by understanding an individual’s interests and needs and build from there to help them identify ways ICT could help them meet these needs. Tech training and support staff for older adults need to be trained in incorporating such an educational component into their regular tech support services. In this case, even when an older client calls for help for one specific issue or skill, while assisting the targeted issue, the supporting staff should intuitively guide them through the various other options that one learned skill could be used for and applied to.

Second, service organizations should include assessment of ICT competency, use, and access into their standard intake protocols. We recognize that customization is difficult to implement in an institution or an organization serving a large number of clients or during times of crisis such as the onset of the COVID-19 pandemic. Therefore, we suggest that tech support services can consider ways to prospectively assess people’s technology access and computer competence on intake and with periodic updates, like other components of a care plan.

Third, we encourage tech support services to also offer available training and resources to clients’ social service providers to give them the necessary skills and tools to help older adults navigate ICT devices and ICT learning experiences. In terms of policy, we call for training for home healthcare workers to provide tech support and teaching skills. Additional funding would be required to pay workers with these new skills, which would cause a necessary and desirable increase in their wages. Further funding should also go to older adult centers, public libraries, religious institutions, and other community-based programs to develop or bring in adequate programs to help older members of their communities get the skills and support they need to become internet users.

Fourth, as suggested by the narratives of some participants who claimed that internet use was just unnecessary and non-usage from the participants with extreme health challenges (including those from the palliative care), some from the current cohort of older adults may just prefer an offline lifestyle or do not have the capacity to learn and utilize a new skill. Therefore, information and social interaction must continue through multiple modalities for older adults and, we would argue, for populations of all ages with all kinds of ICT access barriers.

### Limitations and strengths

This study is not without limitations. First, any recipient of the tech service programs in our partner organization was eligible to participate in the research. However, due to patient confidentiality guidelines at the studied service organization, our researchers were not allowed to recruit participants directly but through word of mouth by the organization’s service staff. Therefore, the number of participants reached remains unknown and the recruitment might have been biased toward individuals who had more success with their devices. It is reasonable to assume that those who did not utilize the tech devices or services were less inclined to participate. Secondly, since the participants had no choice of what device they were offered, we were not able to compare their preferences between devices. Third, the sample is rather small in this research. Future research may benefit from a larger research sample, which can generate more statistically meaningful findings.

Despite these limitations, this study provides unique insight into older adults’ experiences with ICT during the pandemic. A key strength of the research is the diversity of the research sample, which encompasses a broad range of experiences by race, ethnicity, age, educational background, and health characteristics. Importantly, this study uniquely focused on older adults’ technology support and training services during the pandemic. The research findings serve as additional evidence for future tech support and training design for older adults during the ongoing COVID-19 crisis and beyond.

## Data availability statement

The original contributions presented in this study are included in the article/supplementary material, further inquiries can be directed to the corresponding authors.

## Ethics statement

The studies involving human participants were reviewed and approved by the CUNY Hunter College Institutional Review Board. The patients/participants provided their written informed consent to participate in this study.

## Author contributions

All authors listed have made a substantial, direct, and intellectual contribution to the work, and approved it for publication.

## Footnotes

1 The participant reporting suicidal ideation was immediately reported to the service organization, who was familiar with the person’s situation and provided additional clinical support.

## References

[B1] Andreadis A. Zambon R. Parlangeli O. (2021). TV as an experience conveyer for better acceptance of ICT services by older adults. *Univ. Access Inf. Soc.* 20 359–374. 10.1007/s10209-020-00731-w

[B2] Bakardjieva M. (2005). *Internet society: The internet in everyday life.* Thousand Oaks, CA: Sage Publications Ltd.

[B3] Barnard Y. Bradley M. Hodgson F. Lloyd A. (2013). Learning to use new technologies by older adults: Perceived difficulties, experimentation behavior and usability. *Comput. Hum. Behav.* 29 1715–1724. 10.1016/j.chb.2013.02.006

[B4] Chan M. Haber S. Drew L. Park D. (2016). Training older adults to use tablet computers: Does it enhance cognitive function? *Gerontologist* 56 475–484. 10.1093/geront/gnu057 24928557 PMC4873760

[B5] Choi N. G. DiNitto D. M. Lee O. E. Choi B. Y. (2020). Internet and health information technology use and psychological distress among older adults with self-reported vision impairment: Case-control study. *J. Med. Internet Res.* 22:e17294. 10.2196/17294 32490851 PMC7301257

[B6] Crouch E. Gordon N. P. (2019). Prevalence and factors influencing use of internet and electronic health resources by middle-aged and older adults in a us health plan population: Cross-sectional survey study. *JMIR Aging* 2:e11451. 10.2196/11451 PMC6715345 31518256

[B7] Faverio M. (2022). *Share of those 65 and older who are tech users has grown in the past decade.* Washington, DC: Pew Research Center.

[B8] Francis J. Ball C. Kadylak T. Cotten S. R. (2019). “Conceptualizing technology adoption and digital inequalities,” in *Ageing and digital technology: Designing and evaluating emerging technologies for older adults*, eds Neves B. B. Vetere F. (Berlin: Springer), 35–49.

[B9] Freeman S. Marston H. R. Ross C. Morgan D. Wilson G. Gates J. (2022). Progress towards enhanced access and use of technology during the COVID-19 pandemic: A need to be mindful of the continued digital divide for many rural and northern communities. *Healthc. Manage. Forum* 35 286–290. 10.1177/08404704221108314 35855623 PMC9301350

[B10] Gell N. M. Rosenberg D. E. Demiris G. LaCroix A. Z. Patel K. V. (2015). Patterns of technology use among older adults with and without disabilities. *Gerontologist* 55 412–421. 10.1093/geront/gnt166 24379019 PMC4542705

[B11] González-Rivera C. Finkelstein R. (2021). *Meaningful access: Investing in technology for aging well in New York city.* New York, NY: Brookdale Center for Healthy Aging.

[B12] Hänninen R. Taipale S. Luostari R. (2020). Exploring heterogeneous ICT use among older adults: The warm experts’ perspective. *New Media Soc.* 23 1584–1601. 10.1177/1461444820917353

[B13] Harris M. Blocker K. Rogers W. (2022). Older adults and smart technology: Facilitators and barriers to use. *Front. Comput. Sci.* 4:835927. 10.3389/fcomp.2022.835927

[B14] Hecker I. Spaulding S. Keuhn D. (2021). *Digital skills and older worker: Supporting success in training and employment in the digital world.* Washington, DC: Urban Institute.

[B15] Hunsaker A. Nguyen M. H. Fuchs J. Djukaric T. Hugentobler L. Hargittai E. (2019). “He explained it to me and I also did it myself”: How older adults get support with their technology uses. *Socius* 5:2378023119887866.

[B16] Hunsaker A. Nguyen M. H. Fuchs J. Karaoglu G. Djukaric T. Hargittai E. (2020). Unsung helpers: Older adults as a source of digital media support for their peers. *Commun. Rev.* 23 309–330. 10.1080/10714421.2020.1829307

[B17] Kakulla B. (2021). *Personal tech and the pandemic: Older adults are upgrading for better online experiences.* Available online at: https://www.aarp.org/research/topics/technology/info-2021/2021-technology-trends-older-americans.html-CMP=RDRCT-PRI-TECH-040721/?cmp=RDRCT-907b618d-20210416 (accessed December 20, 2022).

[B18] Lafontaine C. Sawchuk K. (2015). “Accessing InterACTion: Ageing with technologies and the place of access,” in *Human aspects of IT for the aged population. Design for aging*, eds Zhou J. Salvendy G. (Cham: Springer), 210–220. 10.1007/978-3-319-20892-3_2

[B19] Lam K. Lu A. Shi Y. Covinsky K. (2020). Assessing telemedicine unreadiness among older adults in the United States during the COVID-19 pandemic. *JAMA Inter. Med.* 180 1389–1391. 10.1001/jamainternmed.2020.2671 32744593 PMC7400189

[B20] Leukel J. Schehl B. Sugumaran V. (2021). Digital inequality among older adults: Explaining differences in the breadth of Internet use. *Inf. Commun. Soc.* 16 139–154.

[B21] Llorente-Barroso C. Kolotouchkina O. Mañas-Vinegra L. (2021). The enabling role of ICT to mitigate the negative effects of emotional and social loneliness of the elderly during the COVID-19 pandemic. *MDPI* 18:8. 10.3390/ijerph18083923 33917966 PMC8068368

[B22] Mills J. Fox J. Damarell R. Tieman J. Yates P. (2021). Palliative care providers’ use of digital health and perspectives on technological innovation: A national study. *BMC Palliat. Care* 20:124. 10.1186/s12904-021-00822-2 34364379 PMC8349145

[B23] Nordin S. Sturge J. Ayoub M. Jones A. McKee K. Dahlberg L. (2021). The role of information and communication technology (ICT) for older adults’ decision-making related to health, and health and social care services in daily life-a scoping review. *Int. J. Environ. Res. Public Health* 19:151. 10.3390/ijerph19010151 35010408 PMC8750227

[B24] Olsson T. Viscovi D. (2018). Warm experts for elderly users: Who are they and what do they do? *Hum. Technol.* 14 324–342. 10.17011/ht/urn.201811224836

[B25] Ostherr K. Killoran P. Shegog R. Bruera E. (2016). Death in the digital age: A systematic review of information and communication technologies in end-of-life care. *J. Palliat. Med.* 19 408–420. 10.1089/jpm.2015.0341 26713368 PMC4827321

[B26] Perrin A. Atske S. (2021). *7% of Americans don’t use the internet. Who are they?* Washington, DC: Pew Research Center.

[B27] Portz J. Ford K. Bekelman D. Boxer R. Kutner J. Czaja S. (2020). “We’re taking something so human trying to digitize”: Provider recommendations for mHealth in palliative care. *J. Palliat. Med.* 23 240–247. 10.1089/jpm.2019.0216 31526220 PMC6987733

[B28] Quan-Haase A. Martin K. Schreurs K. (2016). Interviews with digital seniors: ICT use in the context of everyday life. *Inf. Commun. Soc.* 19 691–707.

[B29] Quan-Haase A. Williams C. Kicevski M. Elueze I. Wellman B. (2018). Dividing the grey divide: Deconstructing myths about older adults online activities, skills, and attitudes. *Am. Behav. Sci.* 62 1207–1228.

[B30] Reneland-Forsman L. (2018). ‘Borrowed access’–the struggle of older persons for digital participation. *Int. J. Lifelong Educ.* 37 333–344.

[B31] Russell D. W. (1996). UCLA loneliness scale (Version 3): Reliability, validity, and factor structure. *J. Pers. Assess.* 66 20–40. 8576833 10.1207/s15327752jpa6601_2

[B32] Santana-Mancilla P. C. Anido-Rifón L. E. (2017). The technology acceptance of a TV platform for the elderly living alone or in public nursing homes. *Int. J. Environ. Res. Public Health* 14:617. 10.3390/ijerph14060617 28594386 PMC5486303

[B33] Schlomann A. Even C. Hammann T. (2022). How older adults learn ICT-guided and self-regulated learning in individuals with and without disabilities. *Front. Comput. Sci.* 3:803740. 10.3389/fcomp.2021.803740

[B34] Sixsmith A. Horst B. R. Simeonov D. Mihailidis A. (2022). Older people’s use of digital technology during the COVID-19 pandemic. *Bull. Sci. Technol. Soc.* 42 19–24. 10.1177/02704676221094731 PMC9038938 38603230

[B35] Smith A. (2014). *Older adults and technology use.* Washington, DC: Pew Research Center.

[B36] Van Deursen A. J. (2020). Digital inequality during a pandemic: Quantitative study of differences in COVID-19–related internet uses and outcomes among the general population. *J. Med. Internet Res.* 22:e20073. 10.2196/20073 32750005 PMC7446757

[B37] Van Deursen A. Dijk V. (2014). The digital divide shifts to differences in usage. *New Media Soc.* 16 507–526. 10.1177/1461444813487959

[B38] Van Deursen A. Helsper E. (2015a). A nuanced understanding of internet use and non-use amongst the elderly. *Eur. J. Commun.* 30 171–187. 10.1177/0267323115578059

[B39] Van Deursen A. Helsper E. (2015b). The third-level digital divide: Who benefits most from being online. *Commun. Inf. Technol. Annu.* 10 29–52. 10.1108/S2050-206020150000010002

[B40] Vaportzis E. Clausen M. G. Gow A. J. (2017). Older adults perceptions of technology and barriers to interacting with tablet computers: A focus group study. *Front. Psychol.* 8:1687. 10.3389/fpsyg.2017.01687 29071004 PMC5649151

[B41] Wang C. Wu C. (2021). Bridging the digital divide: The smart TV as a platform for digital literacy among the elderly. *Behav. Inf. Technol.* 41 1–14. 10.1080/0144929X.2021.1934732

[B42] Witte J. Mannon S. (2010). *The internet and social inequalities.* Oxford: Routledge. 10.4324/9780203861639

[B43] Yap Y. Y. Tan S. H. Choon S. W. (2022). Elderly’s intention to use technologies: A systematic literature review. *Heliyon* 8:e08765. 10.1016/j.heliyon.2022.e08765 35128090 PMC8800037

